# Strategic Management of Early Childhood Caries in Thailand: A Critical Overview

**DOI:** 10.3389/fpubh.2021.664541

**Published:** 2021-06-11

**Authors:** Thanya Sitthisettapong, Parinda Tasanarong, Prathip Phantumvanit

**Affiliations:** Faculty of Dentistry, Thammasat University, Patumthani, Thailand

**Keywords:** dental caries, caries management, oral health, public health, Thailand, early childhood caries

## Abstract

The aim of this report was to advocate early childhood caries (ECC) and share strategic management in Thailand, despite over two decades of free Universal Health Coverage including oral healthcare. The recent Thai national oral health survey in 2017 indicates the very high prevalence of ECC, with an average of three carious teeth affected in 53% of 3-year-old children. This is despite the efforts of the Ministry of Public Health that has launched several interventional programs ranging from an upstream policy that prohibits sugar additions in baby formula milk to downstream remediations such as advocating and encouraging toothbrushing with fluoride toothpastes. Nevertheless, ECC is strongly predicated by other key factors including the family and community commitment and participation, as embodied in the current World Health Organization guidelines. These encompass three different tiers of community-level prevention: primary, secondary, and tertiary. Accordingly, the following strategies for ECC management in Thailand should be based at primary care clusters (PCC) in sub-district health centers, with the assistance of inter-professional health teams. These include community education on the importance of deciduous teeth and effective toothbrushing with fluoride toothpaste (primary prevention), regular examination and detection of ECC lesions and early intervention (secondary prevention), insertion of non-invasive preventive restorations using cost-effective atraumatic restorative treatment (ART) or simplified and modified ART (SMART) (tertiary prevention), and, finally, effective follow-up and monitoring systems. It is anticipated that this triple tier approach to ECC management will improve not only the oral health but also the overall children's health.

## Introduction

Early Childhood Caries (ECC) is a major public health problem in Thailand. The newest national oral health survey from Thailand in 2017 indicates the very high prevalence and severity of ECC in the country, with an average of three carious teeth affected in 53% of 3-year-old children (1).

Reports indicate that ECC in Thailand usually develops from the initial white spot lesion to cavitated dentine caries within a rapid time frame of 12 months in very young dentate children (2–5). As these early carious lesions aretite poorly controlled, they progress into larger cavities, leading to further complications such as abscess formation and related oral pathology. Further, it is now clear that ECC affects not only the oral health but also the overall health and quality of life of these pre-school children.

In general, ECC is a multifactorial disease due to both intra-oral and extra-oral environment factors. Primary teeth are particularly prone to ECC due to their anatomy, thin enamel, and large pulp chambers. Additionally, poor oral hygiene in young children is also a major contributory factor for ECC, as this leads to dysbiosis of the oral microbiome and the development of a cariogenic plaque biofilm. In terms of extrinsic etiologic factors, it is clear that increased sugar consumption in early life can significantly increase ECC (6). Further, a low socioeconomic status and poor oral health literacy of the parents/care givers, unhealthy breast feeding, and/or long-term bottle feeding habits, and the fact that in rural Thai populations ECC is traditionally considered as the norm and neglected, all contribute to the disease process. Furthermore, these avoidable consequences of neglect has arisen in Thailand despite a wide network of public dental therapists (dental nurses) and dentists throughout the jurisdiction. Hence, managing the current burden of ECC in Thailand appears to be a major health issue that has been largely ignored but needs immediate remediation. Therefore, this study aimed to discuss the ECC strategic management as developed internationally and applied in Thailand.

## National Oral Health Surveys of Thailand

The most recent quintennial survey of Thailand National Oral Health, based on the WHO pathfinder survey (7), indicates the magnitude of the problem. The survey found that of the caries severity (DFT/dft) in all age groups, the ECC in 5-year-old children was the most severe. Besides, treatment of caries in primary teeth, especially restorations, was only 4% (ft) in 5-year-old children compared with the restorations of permanent dentition, which was 50% (FT) in the 15-year-old age group (Table 1). Such data are critical for healthcare planners, as early detection of ECC in this younger pre-school children would be an indication for early intervention and strategic management of this burgeoning problem.

**Table 1:** Dental caries prevalence and experience of Thai population from 3-year-old children to 89-year-old elderly (adapted from 8th Thailand National Oral Health Survey in 2017) (1).

**Age (years)**	**Prevalence (%)**	**Teeth**	DT (dt^*^)	MT (mt^*^)	FT (ft^*^)	DMFT (dmft^*^)	DFT (dft^*^)
3^*^	52.9	19.9	2.7	0.0	0.1	2.8	2.8
5^*^	75.6	19.4	4.2	0.1	0.2	4.5	4.4
12	52.0	25.6	0.6	0.0	0.7	1.4	1.3
15	62.7	27.7	0.9	0.1	1.0	2.0	1.9
35–44	91.8	28.4	1.1	3.6	1.9	6.6	3.0
60–74	98.5	18.6	1.8	13.3	0.8	15.9	2.6
80–89	99.5	9.9	1.8	21.9	0.3	24.0	2.1

*DT/dt, decayed teeth; MT/mt, missing teeth; FT/ft, filled teeth*.

* *primary teeth*.

**Figure 1:**
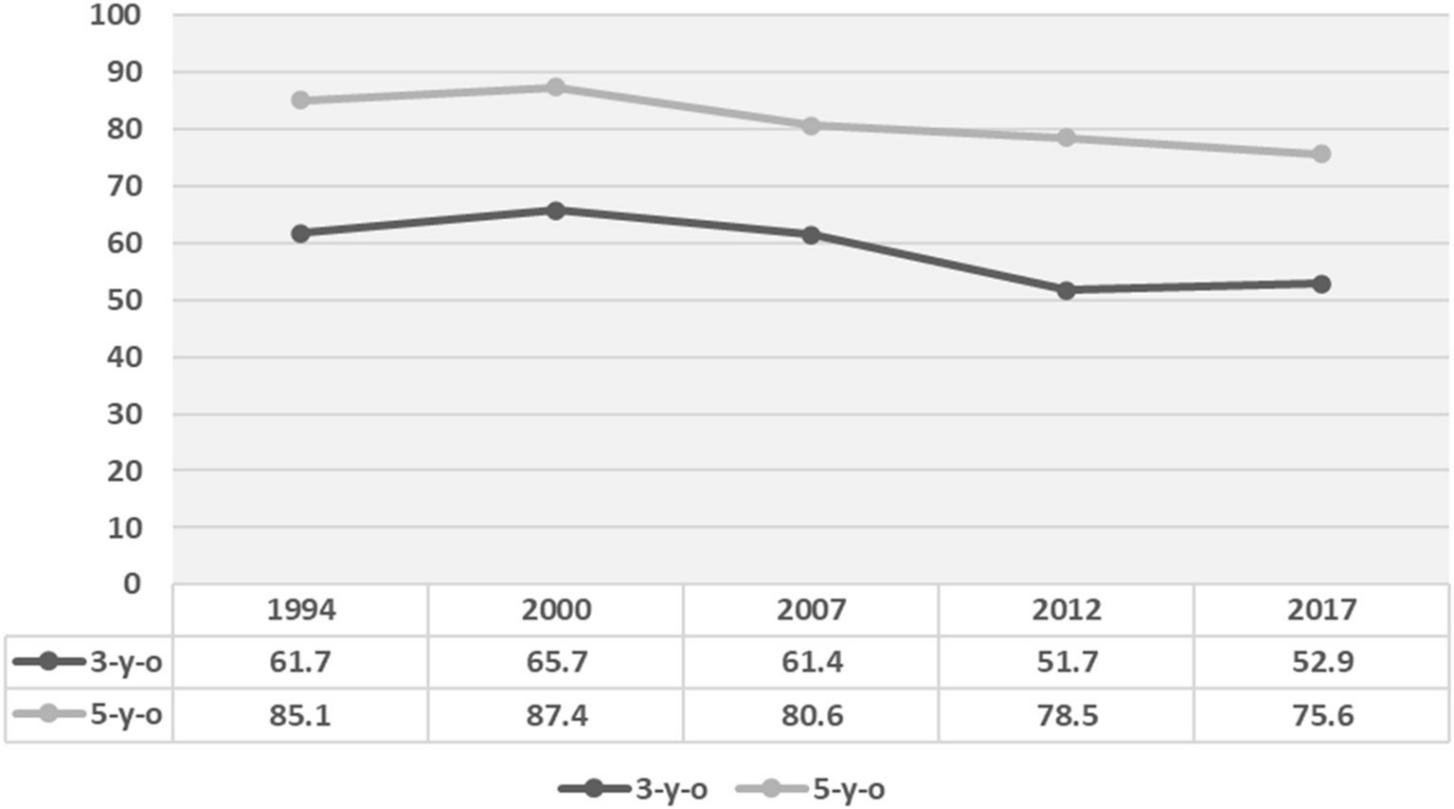
Comparison of dental caries prevalence (%) in primary teeth in the last five National Oral Health Surveys (adapted from 8th Thailand National Oral Health Survey in 2017) (1).

However, the findings also report that, in comparison to the previous surveys, ECC or caries in primary teeth was relatively stable with a marginal reduction over the last decade (Figure 1). This might due to the overall economic developments in the country including the healthcare system. Nevertheless, the overall caries prevalence of ECC in Thailand is unacceptably high compared to international standards. Hence, more serious, proactive strategies for the overall improvement of oral health, general health, and the quality of life of pre-school children countrywide are needed.

## Healthcare Delivery System in Thailand

The accountability for improving the oral health in the Thai population rests with the Bureau of Dental Health. Oral healthcare is mainly provided by dentists and dental therapists working in 899 government hospitals and 9,769 sub-district primary health centers (health promoting hospitals) located countrywide (8). While Thai dentists deliver oral care at primary, secondary, and tertiary levels, dental therapists mainly conduct oral health promotion in community, with simple treatment to children in public dental clinics. However, the survey of dental service utilization in 2015 demonstrated that approximately one-half of the Thai dental patients (46.2%) attended 347 private hospitals and 4,244 private dental clinics, mostly located in Bangkok and urban areas (9, 10). Although the dental service utilization is higher in the urban areas, the dental treatment demand is mainly encountered in the rural areas (1). This implies that an oral health disparity exists between the urban and rural dwellers in the country.

Thailand has implemented Universal Health Coverage (UHC) as a component of their National Health System since 2002 (11), ensuring that every Thai citizen is entitled and have the right to access the essential health services for their health promotion, prevention, treatment, rehabilitation, and palliative care throughout the lifetime. Under UHC, all Thai children age lower than 12 years old can receive free-of-charge oral health promotion and prevention (oral examination, extra- and intra-oral radiographs, fluoride application, and sealant) and dental treatment (filling, pulpal therapy, extraction, and obturator for cleft palate baby) at all dental clinics in the public (government) sector.

As mentioned, the latest national survey (Table 1) demonstrate that more than half of Thai preschool children still suffer from dental caries. Unfortunately, extremely low number of these carious teeth received dental treatment, (0.1 mt and 0.2 ft), figure that has been stable for many decades without any significant increments (1). In addition, another survey in 2015 noted that only 5.8% of preschool children received dental treatment, the smallest proportionate age group that accessed dental service in that year (9). These glaring statistics highlight the issues within the Thai healthcare system as well as other general issues that obviate access to dental treatment for the younger age groups, described above in the introductory narrative. Therefore, oral health promotion, prevention, and management strategies in preschool children in Thailand should be inclusive of both the family and the community, in addition to the governmental measures.

### Role of the Family

Several studies conducted in Thai preschool children have shown that various behavioral issues of both children and parents such as irregular tooth brushing habits of children inculcated by their parents, sleeping with bottle feeding, up to 30 months, breastfeeding to sleep, and poor dietary habits had significant impact on the prevalence of ECC (2–4, 12). Other systematic reviews have indicated a significant association between sociodemographic factors, such as low family incomes, low level of parent education, and low maternal age, resulted in a high prevalence and incidence of ECC (13). A recent national survey in 2017 also noted that 86.8 and 89.4% of 3- and 5-year-old children, respectively, brushed their teeth in the morning, but only 42.5 and 14.4% were supervised or assisted by their parents. Notable though, parents of children residing in Bangkok and urban areas brushed the teeth of their children more frequently than parents in rural areas. These clearly show that, parents or caregivers are the key persons who should take care of child oral health, establishing good oral health behavior and escorting them for early dental visits.

The above revelations of the oral health surveys and related findings have led the Thai authorities to promote oral health literacy among families through parental education. These include education on the importance of primary dentition and its impact on the child's quality of life, as well as motivation to introduce early tooth brushing with appropriate fluoridated toothpastes immediately after tooth eruption. It is known that fluoride toothpaste is the most cost-effective homecare method of dental caries prevention in children who are at high risk for caries (14). These information literacy programs reach most Thai families *via* multimedia, such as the television, radio broadcasts, and internet websites. Yet, practical onsite intervention with effective hands-on training remains the mainstay of educating both parents and children.

### Role of the Community

A number of interventional focal points are involved in the management of ECC at the community level. These include promoting early dental visits in community health programs (15) and early tooth brushing, organized at the Well Baby Clinics (WBC), especially during child's vaccination visits. Some of the features of the latter community programs include (i) oral examination of each child and caries risk assessment performed by dental staff, (ii) training of mothers to detect dental plaque, (iii) practicing hands-on tooth brushing of their child's teeth, (iv) imparting a knowledge of appropriate dietary habits for child's oral health, and (v) fluoride varnish (5% sodium fluoride) application for children at high caries risk. Concurrently, similar oral health education and guidance should be conducted in Antenatal Clinics (ANC). Moreover, pregnant woman with oral disease needs to be referred for dental evaluation and treatment at second trimester (16), as oral health status of the mother has an impact on imitating the transmission of cariogenic organisms to the child. Unfortunately, these procedures have been inconsistently performed by most rural ANCs and WBCs. Subsequently, health promotion activities must be included in day care centers/nursery schools, and these must be delivered by dental therapists and trained village health volunteers nationwide.

Another community initiative called “sweet enough network” was established, with the support of the Thai Health Promotion Foundation and a group of pediatricians, dentists, nutritionists, and independent academics in 2002 to minimize ECC and obesity (17). This network aims to educate parents that ECC is mainly due to excessive consumption of sweetened foods and sugary beverages. Also, the Ministry of Public Health announced a national policy in 2004 for sugar-free baby formulas, for those weaning off breastfeeding (18). Under this network, “sweet enough school” has been promoted in many parts of the country to not introduce soda drinks and crunchy snacks at nursery schools/day-care centers. It is believed that the lower rate of ECC in 3-year-old children noted in the last three consecutive national oral health surveys was partly achieved through the latter “sweet enough network” initiative (Figure 1).

## Strategies for Prevention of ECC

According to WHO Expert Consultation on Public Health Intervention against ECC, recommendations for health promotion and management should be based on three levels of community prevention, viz: primary, secondary, and tertiary prevention (19).

### Primary Prevention

Promoting healthy behavior is the cornerstone of primary prevention, which begins with the mother during her antenatal period. At this stage, all pregnant women should be provided instructions on good oral hygiene practice, for instance, twice daily tooth brushing and avoiding cariogenic diets (20). During the postnatal period, mothers should initiate breastfeeding until the child reaches the age of 6 months, which could be prolonged for up to 2 years (21). Moreover, throughout early years of life, sugar should not be added to a child's food or drink (22). Children should also wean from bottle feeding, at 12–24 months, and avoid consuming fermented carbohydrate containing liquids while bottle feeding or no-spill training cups (23).

Also, home visits should be paid by dental professionals where they train the parents and caregivers on how best to examine for dental plaque of the primary dentition, as well as white spot lesions of incipient caries. In Thailand, the recommend technique is “lift the lip” campaign followed by provision of hands-on toothbrushing and plaque removal training (24).

Children should be brought for their first dental examination as soon as the first tooth erupt, either during their general medical checkup or during the routine vaccination visits. Appropriate and effective, twice daily toothbrushing with fluoride (1,000 ppm) toothpaste should be universally available for all children. When adopted, these measures appear to have led to salutary benefits in reducing ECC. For instance, a recent randomized control study of Thai children aged 0.5–1.5 years, where health education and hands-on training in toothbrushing were provided (with the assistance of the mothers), and triennial monitoring, reported a significant 2.5 times reduced incidence of ECC after 1 year (25). In contrast, another similar randomized control trial reported that although dental health education to parents or caregivers significantly improved oral hygiene practices, such as toothbrushing activities and feeding behavior, this was still inadequate in preventing ECC increments (26).

### Secondary Prevention

Early detection of primary signs of dental caries, such as white spot lesions, should be performed by dental professionals or other well-trained healthcare professionals during the first visit of children to a dental clinic or to a community primary health center. This should be combined with the application of fluoride varnish, used in Thailand for over a decade, both in primary health centers and in dental clinics. A cohort study in Thailand in 2009 reported a 30% reduction of ECC in children younger than 3 years due to fluoride varnish application (27). Moreover, it appears that silver diamine fluoride (SDF; 38%) is more efficacious than fluoride varnish in arresting ECC, as reported in a systematic review where the former effectively arrested the progression of cavitated carious lesions in enamel and dentine of the primary dentition (28). Recently, another randomized control study in Thailand showed that SDF is twice as better in arresting dentine caries in young children compared with fluoride varnish when applied biannually (29).

### Tertiary Prevention

Tertiary prevention aims at controlling disease progression and restoration of the functionality of teeth through simple interventions such as atraumatic restorative treatment (ART) and simplified and modified ART (SMART) using glass ionomer cements. It is now known that glass ionomers used in ART have comparable retention to that of the fluoride releasing composite resins. In a 1-year randomized control study in Thailand, using the above technology and partial caries removal demonstrated that, in primary teeth with class I or II cavities restored with either materials, it showed 100% pulpal survival in radiographic examinations (30).

ART is a simple, straightforward, procedure where dentinal caries is selectively removed with a hand instrument and the deficit restored with a fluoride releasing high viscous glass ionomer cement (31). It is known that their mechanical properties are ideal for this purpose due to the firm chemical bonding with both the enamel and dentine, thermal expansion comparable to that of the tooth structure, biocompatibility, good fluoride releasing ability, and low moisture sensitivity (32). In addition, SMART has been developed as an improved extension of ART, entails partial caries removal (selective removal up to soft dentine) (33), and restores the carious cavity with a capsulated high viscous glass ionomer (34, 35). Both ART and SMART are ideal for field setting as tertiary management tools for ECC.

## Discussion

The healthcare system in Thailand is grounded on the principle of free universal health coverage, with emphasis on the primary care clusters (PCC) that are focused on the family unit and its well-being (11). The PCC so constituted will engage family physicians at the peripheral and sub-district health units assisted by a team of nurses, dental therapists, and other health professionals working together with the “village health volunteers” who overarch this defined community. Although dentists are currently not included in this team, it is likely that in the near future, the PCC will include a family dentist as a member of the latter inter-professional health complex.

The critical importance of the inter-professional healthcare in such PCC cannot be over-emphasized. Dental therapists have a significant role to play here as they could assist and support the dentists in the public sector and other professional team members of the cluster. In addition, they could work as an intra-professional unit and not only collaborate in the management of ECC and oral health but also oversee the general health of the population subgroups.

Early dental experiences of children in formative years are highly likely to modulate their subsequent adult dental behavior (36). Systematic review of current restorative treatment of ECC demonstrated that some ECC cases were treated under general anesthesia; however, this is expensive and traumatic (37). Hence, interventions and management of ECC need to be conducted with gentle care using non-invasive approaches mentioned above to gain the children's trust and for the latter to cultivate good dental attitudes and amicable visits to the dentists throughout his/her lifetime.

In terms of active intervention in ECC management, the Thai approach is for routine early oral examination during home visits or at primary health centers during the childhood vaccination period. These visits are exploited to initiate and deliver oral healthcare for young children at the earliest period of their life. At this stage, as an introductory intervention measure, painless preventive dental procedures such as oral examination, fluoride varnish application, or even sealants application are encouraged as introduction to “dentistry”. Dentine caries lesions are managed by non-invasive, painless, dentistry such as ART and SMART, delivered by either the dental therapist or the dentist. Such approaches have been popularized in Thailand by slogans such as “no injection, no drill and no pain” procedures. These techniques are implemented with the partial caries removal techniques based on the conservative preservation of tooth tissues, aimed toward the protection of healthy pulp tissues (33, 38).

Therefore, at a patient level, ECC management should be limited to control of the disease through preventive and non-invasive measures that are pain-free (39). Keeping such objectives in mind the Ministry of Public Health of Thailand launched in 2018 “The first miracle 1,000 days of life” campaign with the aim of nurturing a caries-free child population, who are healthy during the critical developmental years (40). Such a program, which has been successfully implemented in Brazil, has also an oral health component similar to the Thai program (41).

As mentioned, the importance of inter-professional collaboration in ECC management and subsequent healthcare delivery must not be overlooked. This could be initiated at the primary health centers and subsequently proceed through various stages, mainly in the community hospitals. For example, caries risk assessment for non-dental healthcare providers created by AAPD (American Academy of Pediatric Dentistry) (42) could be modified for this purpose, as these could be implemented by physicians, nurses, or other health workers simply through observation and interviews. Thereafter, children with high caries risk could be referred for appropriate oral health services in contiguous or area dental clinics. An additional advantage of this is that dentists or dental therapists can work with physicians or pediatricians and nutritionists to control both ECC and non-communicable diseases (NCD) such as diabetes and obesity.

Last but not least, improving oral health literacy in the country should be a major strategic goal underpinning the whole exercise. The importance of this is clearly seen in the statistic that only 5.8% of Thai children receive oral health services annually (9). It is anticipated that pro-active oral health services at the domestic day-care centers by PCC dental team may increase dental utilization, especially with ART/SMART.

The foregoing optimal ECC management strategy, operationalized within the free universal healthcare system of Thailand, not only will control ECC but also can lead to the overall improvements of child health, in general, for years to come.

## Data Availability Statement

The original contributions presented in the study are included in the article/supplementary material, further inquiries can be directed to the corresponding author/s.

## Author Contributions

TS, PT, and PP conceived the manuscript, did the searches, and wrote the draft of the manuscript. PP contributed to the discussion. TS and PP critically revised the manuscript. PT rearranged the references. All authors contributed to the article and approved the submitted version.

## Conflict of Interest

The authors declare that the research was conducted in the absence of any commercial or financial relationships that could be construed as a potential conflict of interest.
